# 

**Published:** 1913-11-08

**Authors:** 


### CONTENTS.

| | PAGE |
|---|---|
| **The New Mental Board of Control** ... ... | 127 |
| **Punishment in Poor-Law Infirmaries** ... ... | 128 |
| **Hospital and Institutional News,** including— ... | 129 |
| New Commissioners Under the Mental Deficiency Act—The Insured and Dr. Dimock—Scarlet Fever Accommodation—Reading for Workhouse Inmates—The Hampstead Hospital's Motor Ambulance—Another Hospital for Sale. | |
| **The Clinical Laboratory** ... ... ... ... ... | 135 |
| The Examination of the Stomach Contents. | |
| **Practitioners and Nursing Homes: A Suggested Reform** ... ... ... ... | 137 |
| **The Pitfalls of Panel and Private Practice** ... | 138 |
| By F. J. SMITH, M.D., F.R.C.P. | |
| **The Treatment of "Dull" and "Defective" Children** ... ... ... ... ... ... | 140 |
| **London County Council and Tuberculosis** ... | 141 |
| **The Training of Institutional Porters** ... ... | 142 |
| **Report on Chelmsford and Essex Hospital** ... | 143 |
| By SIR HENRY BURDETT, K.C.B., K.C.V.O. | |
| **Public Pharmacists' and Dispensers' Association** | 144 |
| **An Experience in a Special Hospital** ... ... | 145 |
| **The Crisis at Camberwell Infirmary** ... ... | 145 |
| The Committee's Report on the Medical Staff. | |
| **The Alterations at Rochdale Infirmary** ... | 147 |
| **Buildings Contemplated and in Progress** ... | 148 |
| **The Editor's Letter-Box** ... ... ... ... | 149 |
| "A Peep Behind the Scenes"—Forcible Feeding and the Penal Reform League. | |
| **Appointments** ... ... ... ... ... ... ... | 149 |
| **Institutional Needs** ... ... ... ... ... ... | 149 |
| **With the Wounded in Bulgaria** ... ... ... | 150 |
| **The Non-Panel Movement** ... ... ... ... | 152 |
| **Personalia** ... ... ... ... ... ... | 154 |
| **Gifts and Bequests** ... ... ... ... ... | 154 |
| **The Institutional Worker** ... ... (Suppl.) | 1 |
| **Our Bureau of Information** ... ... ,, | 2 |

